# Impact of perioperative fluorouracil, leucovorin, oxaliplatin, and docetaxel delivery on postoperative survival in locally advanced oesophagogastric adenocarcinoma

**DOI:** 10.1007/s10120-025-01643-5

**Published:** 2025-07-14

**Authors:** Keiji Sugiyama, Sacheen Kumar, Asif Chaudry, Nikhil Patel, Pranav Patel, David Cunningham, Naureen Starling, Sheela Rao, Charlotte Fribbens, Ian Chau

**Affiliations:** 1 Gastrointestinal Unit, Department of Medicine, The Royal Marsden Hospital, Downs Road, Sutton, London, Surrey UK; 2 Department of Medical Oncology, NHO Nagoya Medical Center, Nagoya, Aichi Japan; 3 Department of Upper Gastrointestinal Surgery, The Royal Marsden Hospital, London, UK

**Keywords:** Adjuvant therapy, Esophageal cancer, Gastric cancer, Neoadjuvant therapy

## Abstract

**Background:**

Perioperative fluorouracil, leucovorin, oxaliplatin, and docetaxel (FLOT) is the standard of care for locally advanced oesophagogastric adenocarcinoma (LA-OGA) in Western countries. However, completing treatment is challenging for patients, particularly in the postoperative setting. This study investigated the impact of adjuvant chemotherapy (ACT) administration and treatment completion on survival outcomes in patients receiving FLOT.

**Methods:**

Charts of LA-OGA patients treated from 2017 to 2023 were retrospectively reviewed. Survival was analysed using Kaplan–Meier and restricted mean survival time (RMST) analyses, with propensity score matching (PSM) adjustments. Subgroup analyses were stratified by pathological nodal status and tumour regression grade (Mandard TRG). The primary endpoint was 3-year overall survival (OS).

**Results:**

The study included 233 patients, among whom 62.4% completed the full perioperative FLOT regimen and 21% did not receive ACT. After PSM adjustment, 3-year OS for patients who completed and those who did not complete perioperative therapy was 69% and 57%, respectively (*p* = 0.09). The 3-year OS was 81% and 52% for patients who did and did not receive ACT, respectively (*p* = 0.01). In multivariate analysis, completion of perioperative FLOT was independently associated with improved OS (*p* = 0.04). Survival improvement with ACT was observed in the ypN-positive subgroup but not in the ypN-negative subgroup.

**Conclusions:**

Perioperative FLOT administration is recommended as the standard of care for LA-OGA. The survival impact of ACT might be influenced by pathological lymph node metastasis.

**Supplementary Information:**

The online version contains supplementary material available at 10.1007/s10120-025-01643-5.

## Introduction

Oesophagogastric adenocarcinoma (OGA) constitutes a leading cause of cancer-related mortality and a significant global health problem. Standard management of locally advanced (LA)-OGA involves multimodal therapy, including radical resection combined with systemic therapy. In Western countries, perioperative chemotherapy is the standard of care for LA-OGA [1–3]. The fluorouracil, leucovorin, oxaliplatin, and docetaxel (FLOT) regimen constitutes a newer standard regimen [3]. In contrast, either adjuvant therapy [4–6] or perioperative therapy [4, 6–9] is the standard strategy in East Asia.

Despite the high treatment compliance in the neoadjuvant phase of the Medical Research Council Adjuvant Gastric Infusional Chemotherapy (MAGIC) trial [1] and FLOT4 [3] trials—wherein 90% of patients received all allocated cycles—the initiation and completion rates of postoperative chemotherapy were notably lower. Specifically, in the MAGIC and FLOT-4 trials, 65.6% and 60% of patients, respectively, commenced postoperative chemotherapy, and only 41.6% and 46%, respectively, completed all allocated cycles. This poor treatment adherence indicates that treatment delivery may significantly impact postoperative survival, and previous reports have generated inconsistent results [10–12]. As a relatively new therapeutic option, the FLOT regimen has only been widely adopted since 2019; thus, data to assess the association of prognosis with treatment exposure in the context of perioperative FLOT therapy and surgical resection remain sparse.

Recently, the results of the international multicentre prospective observational study SPACE-FLOT were reported, evaluating whether pathological response, defined by tumour regression grade (TRG), could predict the survival benefit of the postoperative component of FLOT in patients who had undergone resection for oesophagogastric cancer [13]. According to this recent study, continuing adjuvant FLOT appeared effective in patients who achieved partial response to neoadjuvant FLOT but showed no benefit in those with either minimal or complete pathological response. Although SPACE-FLOT was not a randomised study that compared neoadjuvant chemotherapy (NAC) alone with perioperative therapy, its findings highlight the potential value of a pathological response-guided approach.

The present study aimed to evaluate the impact of treatment completion and adjuvant chemotherapy (ACT), including subgroup analyses based on pathological nodal status and pathological response on survival outcomes in patients with LA-OGA treated with FLOT.

## Materials and methods

### Study population

We retrospectively screened consecutive OGA patients who underwent curative resection at The Royal Marsden Hospital, UK, between January 2017 and December 2023. The inclusion criteria were as follows: age > 18 years; histologically confirmed advanced oesophageal, gastric, or gastro-oesophageal junction adenocarcinoma; clinical stage cT2–4 and/or N1–3 (Union for International Cancer Control 8th edition); receiving at least one cycle of neoadjuvant FLOT therapy; Eastern Cooperative Oncology Group (ECOG) performance status 0–2; and adequate organ function, including bone marrow, renal, and hepatic functions. Patients treated with more than four cycles of FLOT in the preoperative phase and non-FLOT regimen such as FOLFOX in the postoperative phase as well as those who died in the perioperative period were included. The exclusion criteria were as follows: patients with prior anticancer treatment other than neoadjuvant FLOT including radiotherapy; patients with metastatic lesion including positive peritoneal cytology or oligometastatic disease; patients with active synchronous cancer. ACT was defined as the administration of platinum-based cytotoxic chemotherapy after surgery. Postoperative chemoradiotherapy was not considered ACT according to this definition.

The study was conducted in accordance with the principles outlined in the Declaration of Helsinki and was approved by the Committee for Clinical Research at Royal Marsden Hospital (SE1356). As this was a retrospective analysis of medical records, obtaining informed consent from individual patients was not required. This study was reported according to the Strengthening the Reporting of Observational Studies in Epidemiology (STROBE) guidelines.

### Perioperative therapy and surgical procedures

Clinical staging was determined for all patients by a multidisciplinary team conference (tumour board). The FLOT regimen consisted of four preoperative and four postoperative 2-week cycles, each including 50 mg/m^2^ docetaxel, 85 mg/m^2^ oxaliplatin, 200 mg/m^2^ leucovorin, and 2600 mg/m.^2^ fluorouracil, administered as a 24-h intravenous infusion on Day 1, as described in the FLOT4 trial [3]. All patients initially received perioperative chemotherapy with the intention to administer standard FLOT. Treatment modifications, such as dose reductions or omission of docetaxel due to toxicity or intolerance, were allowed in line with the original FLOT 4 trial protocol [3].

Surgery was typically scheduled four weeks after the final dose of preoperative chemotherapy. At our institution, surgical procedures include oesophago-gastrectomy with two-field (mediastinal and abdominal) lymphadenectomy for distal oesophageal and Siewert type 1 and 2 gastro-oesophageal junction cancers; extended total gastrectomy or oesophago-gastrectomy via a left thoracoabdominal approach for Siewert type 3 gastro-oesophageal junction cancers; and total or distal gastrectomy with D2 lymphadenectomy for gastric cancers.

### Outcomes and data collection

All data, including clinical and pathological stages, pathological findings, and survival outcomes, were obtained from electronic medical records. Treatment exposure was evaluated by examining the number of therapy cycles and the administration of adjuvant therapy.

### Histopathological response evaluation

All resected surgical specimens were assessed by in-house histopathologists specialising in gastrointestinal cancer. The histopathological response to neoadjuvant FLOT was examined using the Mandard criteria [14], which stratifies the response in accordance with TRG levels 1–5 as follows: TRG 1 (complete regression or fibrosis devoid of any detectable tumour cells), TRG 2 (fibrosis accompanied by isolated tumour cells), TRG 3 (mixture of fibrosis and tumour cells with a dominance of fibrosis), TRG 4 (combination of fibrosis and tumour cells where tumour cells are predominant), and TRG 5 (presence of tumour without any signs of regression). In the context of this investigation, pathological response was defined as TRG 1 or TRG 2 in line with published randomised controlled trial using the Mandard criteria [15] and other contemporaneous cohort studies using neoadjuvant FLOT [16, 17]. Importantly, TRG 1 and TRG 2 had similar survival after neoadjuvant chemotherapy [16]. We performed further sensitivity analyses using a trichotomised classification with the approach adopted by the SPACE-FLOT study [13, 18] as follows: TRG 1 (complete responders), TRG 2–4 (partial responders), and TRG 5 (minimal responders).

### Outcome measures

The primary outcome of this study was 3-year overall survival (OS), defined as the time from radical surgery to death from any cause. Three-year recurrence-free survival (RFS) was defined as the time from radical surgery to disease recurrence, progression, initiation of any anticancer treatment beyond the planned FLOT regimen, or death from any cause. Survival probabilities were estimated using the Kaplan–Meier method and compared using the log-rank test. The Cox proportional hazards model was employed to derive hazard ratios (HRs) and their corresponding 95% confidence intervals (CIs). P-value was derived from the log-rank test. In addition to the Kaplan–Meier method, the restricted mean survival time (RMST) at 36 months was used to evaluate survival outcomes.

Survival analyses were conducted for the entire cohort, which was stratified by completion of perioperative therapy and administration of ACT. Subgroup analyses were performed based on pathological nodal status (ypN-positive vs ypN-negative) and TRG (TRG 1–2 vs TRG 3–5). Further TRG sensitivity analyses used the trichotomised classification with the approach adopted by the SPACE-FLOT study.

### Propensity score matching (PSM)

To adjust for potential confounders and minimise selection bias, PSM was performed. The following covariates were included in the propensity score model: age, sex, ECOG performance status (0 vs 1–2), body mass index, weight loss at diagnosis, histological type (differentiated or mixed vs poorly differentiated), mismatch repair (MMR) protein expression status (pMMR vs dMMR vs not assessed), tumour location (oesophagus vs gastro-oesophageal junction vs stomach), clinical T stage (1–2 vs 3–4), clinical N stage (N0 vs N1–3), neutrophil-to-lymphocyte ratio (NLR), serum albumin level, and presence of chronic kidney disease (CKD). Among these, CKD, serum albumin, and age were selected as surrogate indicators of baseline comorbidity burden and physiological reserve, which are known to influence chemotherapy safety, dose adjustments, and treatment adherence. Variables such as NLR and tumour staging were included to reflect disease biology and prognosis. All variables and clinical factors were obtained before commencing neoadjuvant FLOT. Propensity scores were estimated using logistic regression, and patients were matched 1:1 using a calliper size of 0.2. RFS and OS were analysed in the matched cohort. PSM was performed only for the overall cohort to evaluate the impact of perioperative FLOT completion and ACT administration.

Subgroup analyses of the impact of ACT on OS and RFS stratified by pathological lymph node status and pathological response were considered exploratory and were conducted using unmatched data, as applying PSM to these smaller subgroups would have substantially reduced the sample size and statistical power. Similarly, covariate-adjusted survival analyses based on Cox regression were not performed in these subgroups due to the limited number of cases and events. As such, comparisons within these subgroups should be interpreted with caution.

### Statistical analysis

Statistical significance was defined by a two-sided *p*-value < 0.05. Categorical variables were compared using the Chi-square test. Cases with missing data were managed using a listwise deletion approach, excluding any observations with missing values from PSM or multivariate analysis.

Both univariate and multivariate Cox regression analyses were performed to assess the impact of clinicopathological factors on RFS and OS. Key variables included age, ECOG performance status, pathological nodal status, pathological T stage, histological differentiation, R0 resection status, and completion of perioperative FLOT. Variables with *p* values ≤ 0.1 in univariate analyses were included in the multivariate analyses. In addition, post-hoc power analyses were conducted to estimate the statistical power of the survival comparisons in treatment delivery. Power was calculated based on group-specific 3-year OS or RFS rates and sample sizes (Supplementary Material 1). All statistical analyses were conducted using R software, with the EZR graphical interface (The R Foundation for Statistical Computing, Vienna, Austria), which provides a comprehensive suite of biostatistical tools.

## Results

### Patient characteristics

This study included 233 patients, representing 55% of those with OGA who underwent resection during the study period. Patients were excluded for the following reasons: prior treatment for oesophagogastric cancer (n = 13), treatment with non-FLOT therapies (n = 106) or chemoradiotherapy (n = 11), incomplete clinical data (n = 9), and synchronous cancer (n = 9) (Supplementary Material 2). Table 1 summarises patient characteristics for the whole study cohort. Median age of patients was 63 years and 78% of the patients were male. Tumour locations included oesophagus (50.6%), gastro-oesophageal junction (19.7%), and stomach (29.6%). Moreover, 76.7% of patients had clinical T3–T4 stage tumours and 60.9% had clinical regional lymph node metastases.

**Table 1:** Patient characteristics

Characteristics		Patients, n = 233
Age, years, median (range)		63 (26–81)
Sex	Male/female	183 (78.5)/50 (21.5)
ECOG-PS	0/1/2	134 (57.5)/98 (42.1)/1 (0.4)
BMI (kg/m^2^)	< 18.5/18.5–24.9/25.0–29.9/ ≥ 30	6 (2.6)/83 (35.9)/86 (37.2)/56 (24.2)
Histology	Differentiated or mixed/Poorly differentiated	140 (60.1)/93 (39.9)
HER2	Positive/Negative/Not assessed	186 (79.8)/31 (13.3)/16 (6.9)
MMR status (IHC)	Deficient/Proficient/Not assessed	7 (3.0)/188 (80.7)/38 (16.3)
Tumour location	Oesophagus/OGJ/Gastric	118 (50.6)/46 (19.7)/69 (29.6)
Clinical T stage	1/2/3/4	0 (0)/52 (22.3)/150 (64.4)/31 (12.3)
Clinical N stage	0/1/2/3	91 (39.1)/72 (30.9)/65 (27.9)/5 (2.1)

*BMI* body mass index; *ECOG-PS* eastern cooperative oncology group performance status; *HER2* human epidermal growth factor receptor 2; *IHC* immunohistochemistry; *MMR* mismatch repair; *OGJ* oesophagogastric junction

### Treatment exposure

The median number of FLOT cycles administered was eight (range: 1–8), and the mean was 6.8. Supplementary Material 3a shows the proportion of patients receiving each phase of chemotherapy in entire cohort and by type of operation. Supplementary Material 3b shows the distribution of the total number of FLOT cycles administered. Of 233 patients, 221 (94.8%) completed all four cycles of NAC and 184 (79%) commenced ACT. Of the 184 patients who received ACT, 181 (98.4%) continued with postoperative FLOT. The remaining three patients received alternative regimens due to intolerance or hypersensitivity: one each received carboplatin plus capecitabine, cisplatin plus capecitabine, and FOLFOX. Given the small number and context-specific nature of these cases, the ACT group predominantly reflects adjuvant FLOT. These patients were nonetheless included in the "perioperative completion" group, as they received postoperative chemotherapy consistent with the intent of completing systemic perioperative treatment. The overall treatment completion rate for eight cycles of perioperative FLOT was 62.4%, with two patients receiving all eight cycles upfront in the preoperative phase. The reasons for the omission of ACT were not systematically documented due to the retrospective nature of the dataset. Potential contributing factors may have included post-operative complications, poor performance status, or physician/ patient decision.

### Survival outcomes based on perioperative therapy completion

**Fig. 1:**
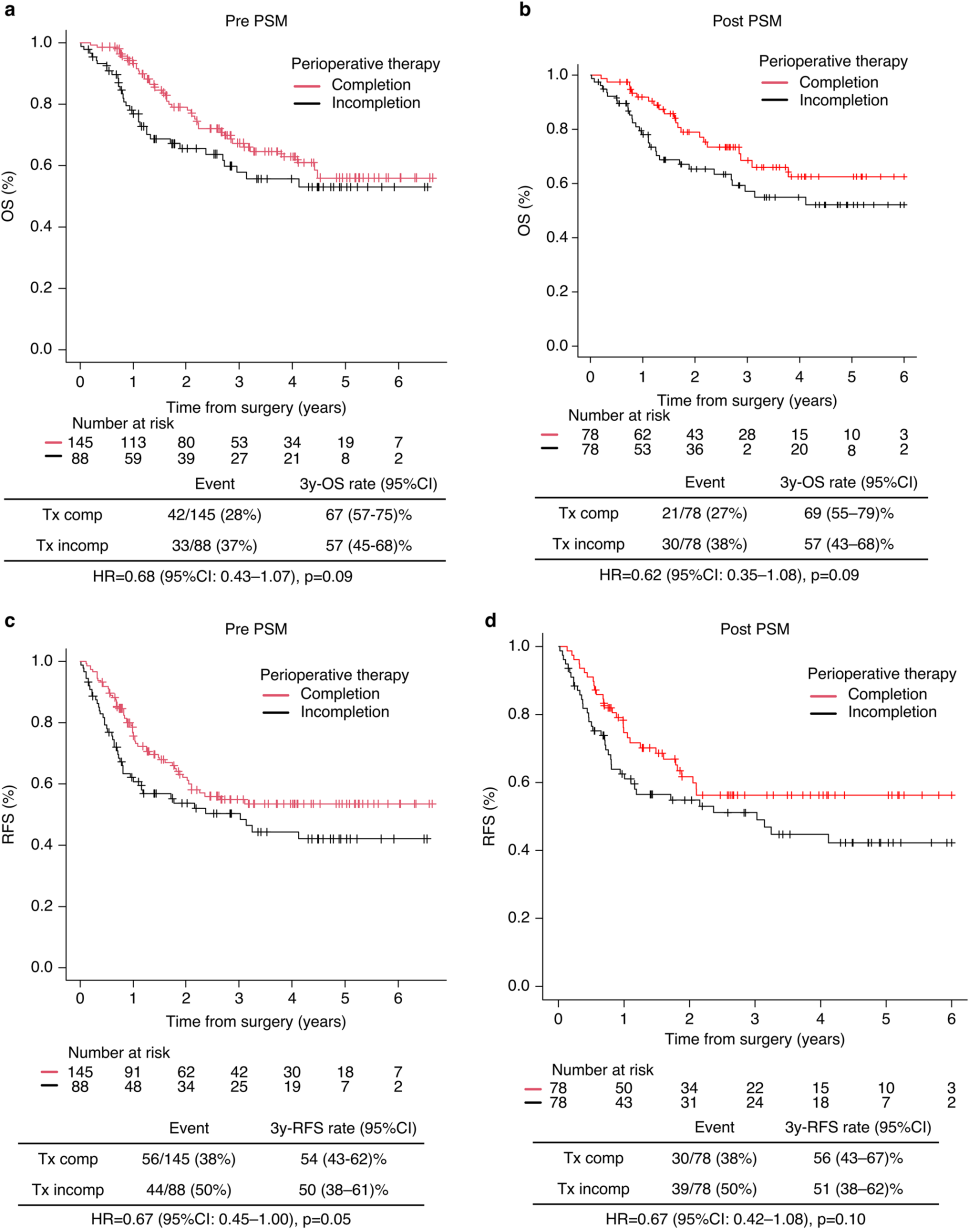
OS and RFS in patients who completed perioperative FLOT vs those who did not. Kaplan–Meier curves before (**a**, **c**) and after (**b**, **d**) propensity score matching. FLOT, 5-fluorouracil, leucovorin, oxaliplatin, docetaxel; OS, overall survival; RFS, recurrence-free survival

The median follow-up time was 25.4 months (range: 0.2–80). Figure 1a shows OS according to perioperative therapy completion. The 3-year OS was 67% (95% CI: 57–75%) in the perioperative therapy completion group and 57% (95% CI: 47–68%) in the non-completion group (*p* = 0.09) The 3-year RFS was 54% (95% CI: 43–62%) and 50% (95% CI: 38–61%) (*p* = 0.05) for patients who completed and who did not complete perioperative therapy, respectively (Fig. 1c). After PSM (Supplementary Material 4a shows baseline characteristics before and after PSM), the 3-year OS and RFS were similarly longer in the perioperative therapy completion group (Fig. 1b, d).

### Survival outcomes stratified by ACT administration

**Fig. 2:**
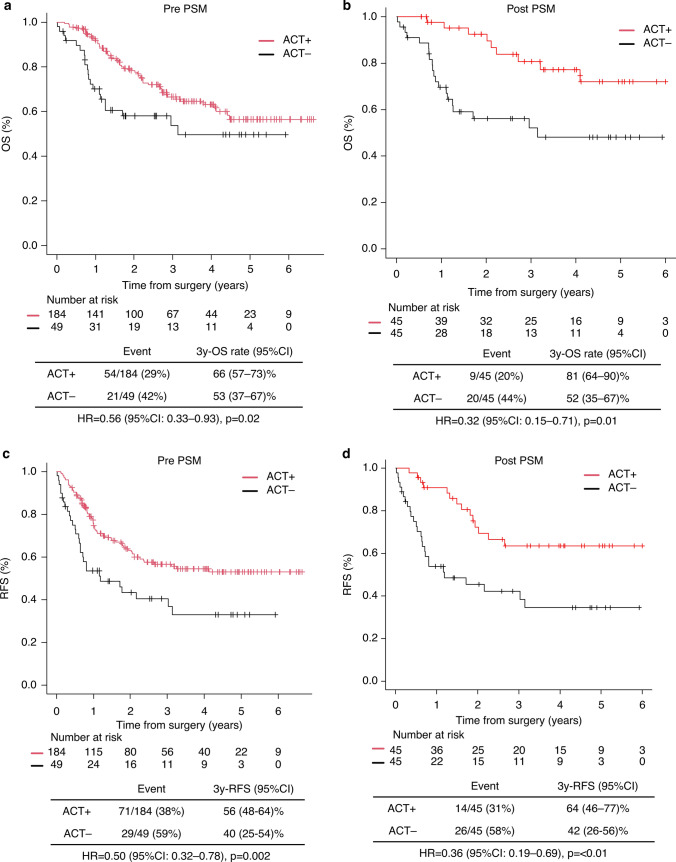
OS and RFS in patients who received adjuvant chemotherapy vs those who did not. Kaplan–Meier curves before (**a**, **c**) and after (**b**, **d**) propensity score matching. OS, overall survival; RFS, recurrence-free survival

The 3-year OS was 66% (95% CI: 57–73%) for patients who received ACT and 53% (95% CI: 37–67%) for those who did not (*p* = 0.02) (Fig. 2a). The 3-year RFS was 56% (95% CI: 48–64%) for patients who received ACT and 40% (95% CI: 25–54%) for those who did not receive ACT ((*p* = 0.002); Fig. 2c). After PSM (Supplementary Material 4a shows baseline characteristics before and after PSM), the ACT group consistently demonstrated longer OS and RFS (Fig. 2b, d).

### Subgroup analysis based on pathological nodal status

**Fig. 3:**
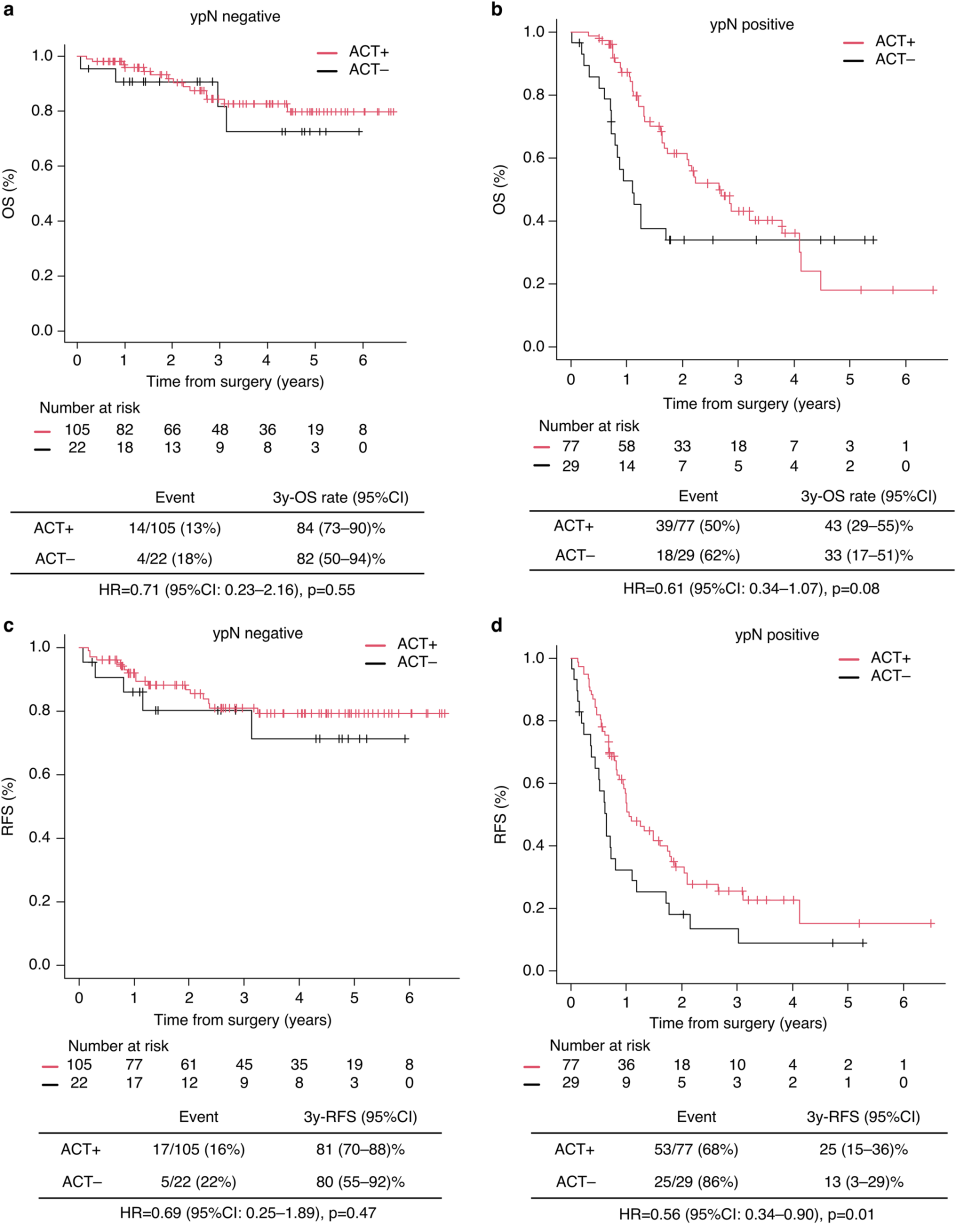
Subgroup analysis of the impact of ACT on OS (**a**, **b**) and RFS (**c**, **d**) stratified by pathological lymph node. OS and RFS in patients who received adjuvant chemotherapy vs those who did not. Survival curves are based on unmatched data. OS, overall survival; RFS, recurrence-free survival

Among the study cohort, 127 patients (54.5%) were pathological node-negative, and 106 (45.5%) were node-positive. Supplementary material 4b shows baseline characteristics according to nodal status. In the pathological node-negative subgroup (ypN0), ACT administration did not significantly impact survival. The 3-year OS was 84% (95% CI: 73–90%) for patients who received ACT versus 82% (95% CI: 50–94%) for those who did not (*p* = 0.55) (Fig. 3a). The 3-year RFS was 81% for patients who received ACT (95% CI: 70–88%) versus 80% (95% CI: 55–92%) for those who did not (*p* = 0.47) (Fig. 3c). Conversely, in the pathological node-positive subgroup (ypN1–3), ACT improved survival. The 3-year OS was 43% (95% CI: 29–55%) for patients who received ACT versus 31% (95% CI: 17–51%) for those who did not (*p* = 0.08) (Fig. 3b). The 3-year RFS was 25% (95% CI: 15–36%) for patients who received ACT versus 13% (95% CI: 3–29%) for those who did not (*p* = 0.01) (Fig. 3d).

### Subgroup analysis based on TRG

**Fig. 4:**
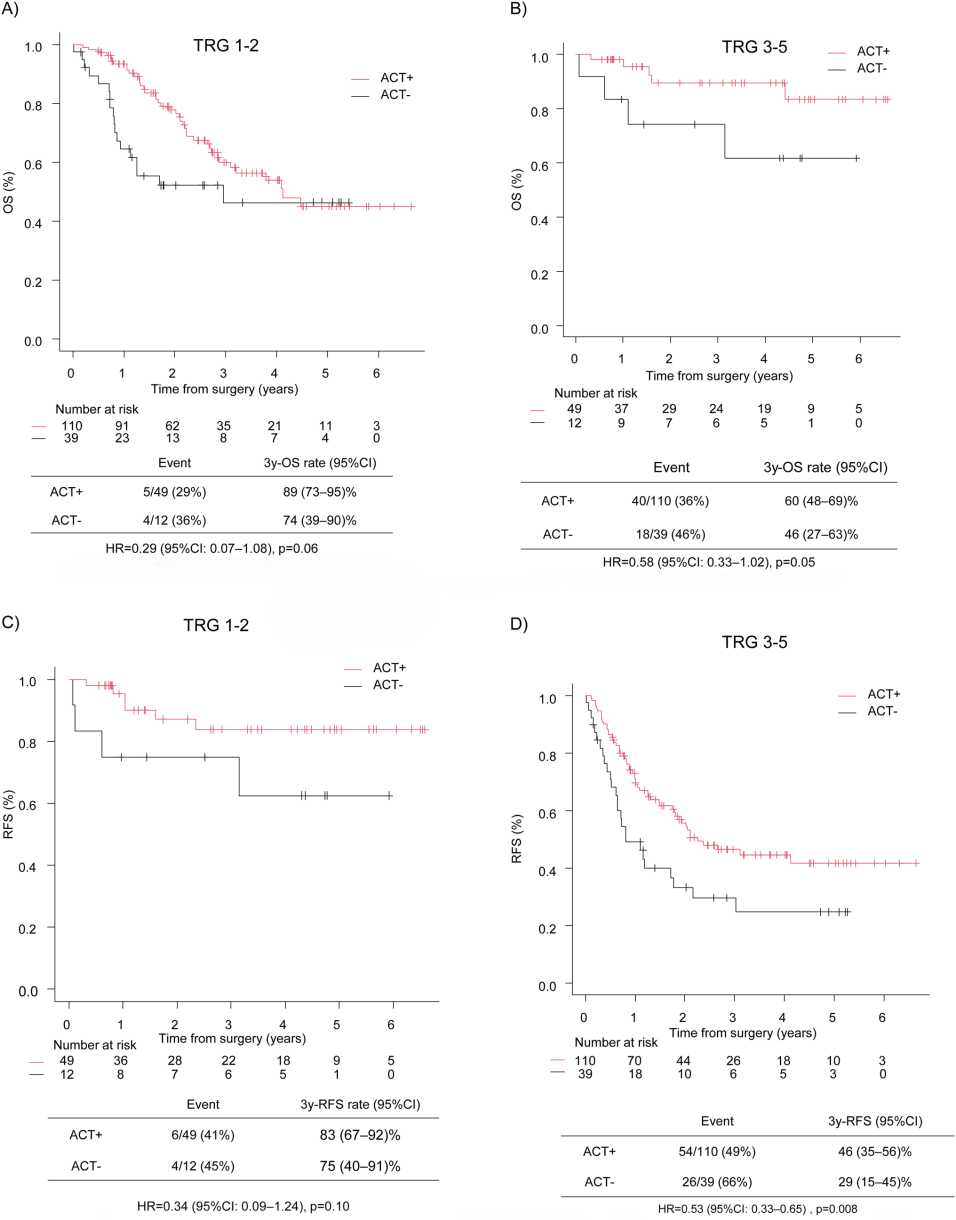
Subgroup analysis of the impact of ACT on OS (**a**, **b**) and RFS (**c**, **d**) stratified by TRG group (1–2 and 3–4). OS and RFS in patients who received adjuvant chemotherapy vs those who did not. Survival curves are based on unmatched data. OS, overall survival; RFS, recurrence-free survival, TRG, tumour regression grade

**Fig. 5:**
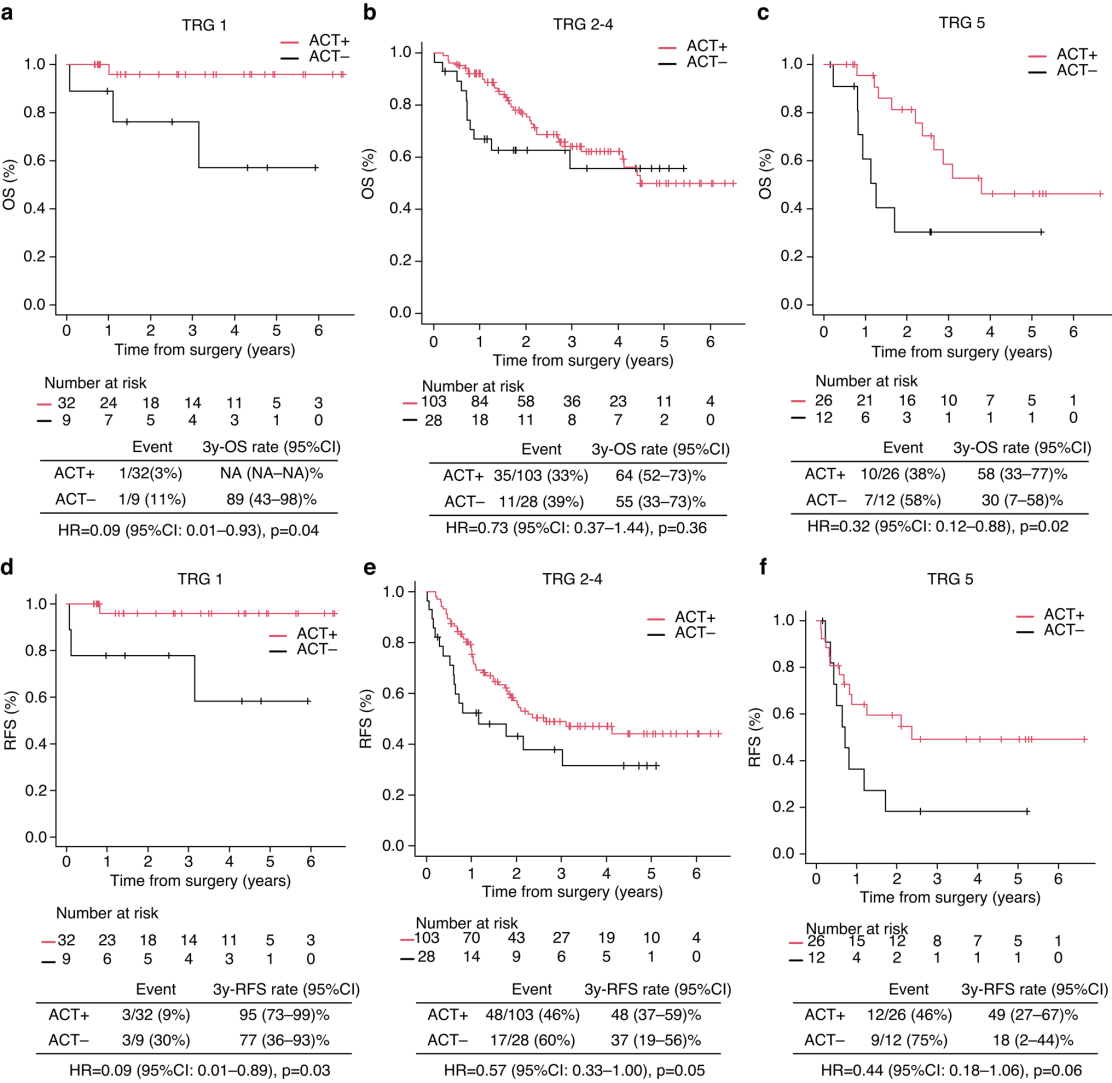
Subgroup analysis of the impact of ACT on OS (**a**, **b**) and RFS (**c**, **d**) stratified by TRG group (1, 2–4 and 5). OS and RFS in patients who received adjuvant chemotherapy vs those who did not. Survival curves are based on unmatched data. OS, overall survival; RFS, recurrence-free survival, TRG, tumour regression grade

Pathological response to neoadjuvant therapy was evaluable in 210 of 233 (90.1%) patients. In the TRG 1–2 subgroup (n = 149), although the differences did not reach statistical significance, patients who received ACT showed numerically better survival outcomes than patients who did not receive ACT. The 3-year OS was 89% (95% CI: 73–95%) for patients who received ACT versus 74% (95% CI: 39–90%) for those who did not (*p* = 0.06) (Fig. 4a). The 3-year RFS was 83% (95% CI: 67–92%) in the ACT group versus 75% (95% CI: 40–91%) in the non-ACT group (*p* = 0.10) (Fig. 4c). Similarly, in the TRG 3–5 subgroup (n = 61), ACT was associated with better survival outcomes. The 3-year OS was 60% (95% CI: 48–69%) in the ACT group versus 46% (95% CI: 27–63%) in the non-ACT group (*p* = 0.05) (Fig. 4b). The 3-year RFS was 46% (95% CI: 35–56%) in the ACT group versus 29% (95% CI: 15–45%) in the non-ACT group (*p* = 0.008) (Fig. 5d).

In our sensitivity analyses of trichomatising pathological responses, 41 (19.5%) were classified as TRG1, 131 (62.4%) as TRG2–4, and 38 (18.1%) as TRG5 (Supplementary Material 4c shows baseline characteristics). In the TRG 1 subgroup (complete responders), ACT was associated with improved survival outcomes. The 3-year OS rate in the ACT group was not estimable due to the limited number of events, whereas it was 89% (95% CI: 43–98%) in the non-ACT group (*p* = 0.01) (Fig. 5a). Similarly, the 3-year RFS was 95% (95% CI: 73–99%) in the ACT group compared with 77% (95% CI: 36–93%) in the non-ACT group (*p* = 0.03) (Fig. 5d). In the TRG 2–4 subgroup (partial responders), no statistically significant differences in survival were observed between groups. The 3-year OS was 64% (95% CI: 52–73%) for patients who received ACT and 55% (95% CI: 33–73%) for those who did not (*p* = 0.29) (Fig. 5b). The 3-year RFS was 48% (95% CI: 37–59%) and 37% (95% CI: 19–56%) in the ACT and non-ACT groups, respectively (*p* = 0.050) (Fig. 5e). In the TRG 5 subgroup (minimal responders), ACT was associated with higher survival rates. The 3-year OS was 59% (95% CI: 34–77%) and 30% (95% CI: 7–58%) in the ACT and non-ACT groups, respectively (*p* = 0.021) (Fig. 5c). Similarly, the 3-year RFS was 49% (95% CI: 28–68%) and 18% (95% CI: 3–44%) in the ACT and non-ACT groups (*p* = 0.061), respectively (Fig. 5f).

### Restricted mean survival time analyses

Supplementary Material 5 shows restricted mean survival time differences at 36 months indicating survival benefits of ACT, especially in patients with pathological node positive status, but not in pathological negative status.

### Univariate and multivariate analyses

Table 2 shows univariate and multivariate analyses. Univariate analysis identified perioperative therapy completion (HR = 0.68, 95% CI: 0.43–1.07, *p* = 0.09), ypN positivity (HR = 5.61, 95% CI: 3.28–9.60, *p* < 0.001), poorly differentiated tumours (HR = 1.70, 95% CI: 1.07–2.67, *p* = 0.02), and R1 resection (HR = 3.42, 95% CI: 1.84–6.36, *p* < 0.001) as significant prognostic factors for OS. Multivariate analysis confirmed that ypN positivity (HR = 4.38, 95% CI: 2.43–7.89, *p* < 0.001) and perioperative therapy completion (HR = 0.62, 95% CI: 0.39–0.98, *p* = 0.04) were independent prognostic factors.

**Table 2:** Univariable and multivariable analyses of overall survival

Variables	OS			
	Univariable		Multivariable	
	HR (95% CI)	*p*-value	HR (95% CI)	*p*-value
ECOG-PS 0 (ref) vs. 1–2	0.86 (0.54–1.36)	0.52		
Age < 65 vs ≥ 65	1.02 (0.65–1.62)	0.90		
ypN negative (ref) vs positive	5.61 (3.28–9.60)	< 0.001	4.38 (2.43–7.89)	< 0.001
ypT0–2 (ref) vs 3–4	3.09 (1.79–5.33)	< 0.001	1.57 (0.86–2.87)	0.13
Non-poorly diff (ref) vs. poorly diff	1.70 (1.07–2.67)	0.02	1.43 (0.89–2.28)	0.13
R0 resection (ref) vs 1	3.42 (1.84–6.36)	< 0.001	1.40 (0.73–2.68)	0.31
Perioperative FLOT completed vs not (ref)	0.68 (0.43–1.07)	0.09	0.62 (0.39–0.98)	0.04

*CI* confidence interval; *Diff* differentiated histology; *ECOG-PS* eastern cooperative oncology group performance status; *HR* hazard ratio; *OS* overall survival; *NAC* neoadjuvant chemotherapy; *Perioperative FLOT* perioperative fluorouracil, leucovorin, oxaliplatin, docetaxel; *Ref* reference

## Discussion

This study evaluated the impact of treatment delivery on postoperative survival in patients with LA-OGA treated with perioperative FLOT therapy and radical surgery. The findings present several important insights into the role of ACT in improving outcomes for these patients.

Completion of perioperative FLOT was associated with improved OS, though this was not statistically significant in the Kaplan–Meier analysis. However, multivariable Cox regression identified it as an independent prognostic factor, highlighting the clinical relevance of maintaining postoperative treatment. Furthermore, the present study demonstrated that ACT administration is associated with improved OS in LA-OGA patients within the perioperative FLOT era. Previously, at least 15 retrospective studies evaluated oncological outcomes in patients treated with NAC alone versus those receiving NAC plus ACT (perioperative therapy) following resection of LA-OGA (Supplementary Material 6) [10–12, 22, 23, 25–32, 35, 36]. Most of these studies included patients who did not receive the FLOT regimen, primarily due to their earlier study periods. Among these, nine studies demonstrated a survival benefit in patients treated with ACT. Although five studies reported no significant association between ACT and improved OS in the entire cohort, subgroup analyses suggested the effectiveness of ACT in specific groups, including ypN-positive patients [10], patients treated with FLOT [31], and patients with R1 resection margins [32]. Recently, the concept of “return to intended oncologic treatment” (RIOT) has emerged in surgical oncology [33]. Previous reports on RIOT in gastric cancer have shown that failure to achieve RIOT (non-RIOT) is associated with poor prognosis [19]. Factors that improve the RIOT rate include the implementation of enhanced recovery pathways [20] and minimally invasive surgical techniques [34]. RIOT is influenced by several factors, such as surgical complications, underlying comorbidities, and physical performance. Additional determinants, such as patient age, socioeconomic status, healthcare access, and quality of surgical care, also play a critical role [19, 33]. These findings underscore the critical need for comprehensive, multidisciplinary supportive care, which has the potential to improve RIOT rates and, ultimately, patient outcomes.

A key clinical question regarding perioperative therapy concerns the role of ACT in specific subgroups defined by pathological factors, such as ypN status and pathological response to NAC. The described relationship between pathological response to NAC and the impact of ACT has been inconsistent in the scientific literature. Two studies demonstrated that the survival benefit of ACT was evident in patients with a favourable histopathological response [12, 35]. In contrast, another report indicated that the benefit was more pronounced in patients with poor histological response to NAC [36]. In our study, the benefit of ACT was observed in both TRG 1–2 and TRG3-5. A previous study [37] analysing 761 cases from two phase III trials [15, 21] showed a similar prognosis for patients with TRG1 and TRG2, with a 3-year OS of 77% for both groups. These findings suggest that pathological response could guide adjuvant therapy decisions, beyond serving as a prognostic marker. However, the SPACE-FLOT study reported a survival benefit of adjuvant FLOT only in patients who achieved partial pathological response to neoadjuvant FLOT. When stratified by tumour regression grade (TRG) using the trichotomisation adopted in SPACE-FLOT (TRG1, TRG2–4, TRG5) in our data, we found that ACT improved RFS but not OS in TRG1, showed no significant benefit in TRG2–4, and improved both OS and RFS in TRG5. These results differ from those of SPACE-FLOT, which reported ACT benefit predominantly in partial responders (TRG2–4). While our data showed apparent benefit in complete and minimal responders, the small subgroup sizes and lack of adjusted analyses preclude definitive conclusions. For example, in the TRG1 group, the value of NLR and BMI differed between patients with or without ACT. The post-hoc power was insufficient to detect the significant difference in TRG2–4. Therefore, any discrepancies in directly comparing our sensitivity analysis data and the SPACE-FLOT study should be interpreted carefully. A recently published study using peri-operative FLOT also showed divergent result to SPACE-FLOT with the most survival benefit from ACT observed in those with minimal histopathological response [17].

Consistent with prior studies involving large cohorts (n = 3449 [10] and n = 4139 [11]), our analysis showed that ACT improves OS in ypN-positive patients but not in ypN-negative patients. Our findings are aligned with these results. Lin et al. highlighted that the prognostic impact of ACT may be influenced by the lymph node ratio (LNR), which is defined as the number of positive lymph nodes divided by the total number of examined lymph nodes [22]. Further, they demonstrated that ACT was associated with improved OS in patients with a higher LNR (9% or greater) but not in those with a lower LNR. These findings suggest that LNR is potentially a more powerful tool than simply assessing the presence or absence of pathological lymph node metastasis for evaluating the impact of ACT following neoadjuvant therapy and curative resection. Conversely, Ballhause et al. reported that ACT did not significantly improve outcomes in the ypN-positive subgroup [23].

The VESTIGE trial, a randomised phase 2 study, enrolled patients with oesophagogastric cancer and high-risk pathological features, including ypN-positive status and/or positive (R1) resection margins [24]. Patients were randomised to continue the same chemotherapy regimen used during NAC or to receive adjuvant immunotherapy with ipilimumab and nivolumab. Notably, 92.3% of these patients had been treated with FLOT in the neoadjuvant setting. The trial revealed significantly worse disease-free survival in the adjuvant immunotherapy arm (HR = 1.55, 95% CI: 1.07–2.25, *p* = 0.02), strongly suggesting the benefits of continuing FLOT in the postoperative setting with high risk pathological features. Our findings in the ypN-positive subgroup align with these results.

In contrast, our results indicate that ACT may not offer significant benefits for patients without lymph node metastases, which highlights the potential for a pathological nodal status-based customised treatment approach in the postoperative setting. Pathological nodal status was previously identified as the most reliable prognostic marker within the perioperative therapy paradigm before FLOT became the standard regimen, as validated by post hoc analyses of multiple phase 3 trial datasets [37, 38]. This finding has now been further confirmed through multivariate analysis in the present study, which included patients treated with FLOT. Overall, these findings highlight the need for further investigations to determine whether de-escalating postoperative therapy is appropriate for ypN-negative patients. However, it should be noted that some ypN-negative patients, such as those with high T stage or poorly differentiated tumours, may still carry a poor prognosis. Therefore, de-escalation strategies should be applied cautiously and ideally guided by comprehensive pathological and clinical risk factors. Monitoring of molecular residual disease (MRD) through postoperative circulating tumour DNA [39] has emerged as a promising method for predicting recurrence and it may help identify patients with truly a low risk of recurrence and refine patient selection for postoperative therapy. Conversely, intensified or modified strategies may be appropriate for ypN-positive or TRG5 patients. Based on current evidence from SPACE-FLOT and VESTIGE, continuing FLOT appears to be the most effective option for ypN positive patients, particularly when a pathological response has been achieved. Integrating pathological and molecular subtyping with MRD assessment could provide critical insights into recurrence risk, enabling more personalised strategies beyond the current uniform approach in LA-OGA. This perspective warrants further investigation in future clinical and translational research.

This study has some limitations. First, as a single-centre retrospective cohort study, it is subject to inherent biases. However, the survival outcomes, R0 resection rate, and treatment delivery appear comparable to those reported in recent phase 3 trials focusing on FLOT, such as AIO-FLOT4 [3] and ESOPEC [40]. This comparison supports the validity and relevance of our outcomes and results. Second, the retrospective nature of the study introduces biases, particularly regarding patient characteristics that influenced the decision to initiate or complete postoperative therapy. We performed PSM to adjust for baseline factors before the initiation of neoadjuvant FLOT, thereby enhancing the validity of our comparisons. However, other clinical factors that could potentially influence the decision to commence ACT, such as surgical complications, length of hospital stay, postoperative weight changes, and functional status after surgery [41, 42], were not included in the PSM. This limitation may have led to an overestimation of the survival benefit associated with perioperative therapy. Third, the median follow-up time of 25 months may be insufficient to robustly estimate 3-year survival outcomes. To address this limitation, we performed additional restricted mean survival time (RMST) analyses at 36 months, which are provided in Supplementary Material 5. These analyses were intended to complement the Kaplan–Meier and Cox regression results. RMST estimates showed consistent survival advantages for both perioperative FLOT completion and ACT administration, with the most pronounced effect observed in the ypN-positive subgroup. However, given their exploratory nature and partial overlap with the primary analysis, these findings should be interpreted with caution. Finally, subgroup analyses stratified by pathological nodal status or pathological response were analysed with an unadjusted and unmatched cohort due to limited sample size and the number of events; therefore, findings of the impact of ACT in each category defined with TRG should be interpreted carefully.

In conclusion, treatment completion and perioperative FLOT therapy are recommended for LA-OGA patients. Data from the completed phase III MATTERHORN study, which has recently reported a significant event-free survival benefit with the addition of durvalumab to FLOT, may provide further insights [43]. However, in real-world settings, postoperative chemotherapy after major surgical resection is challenging for patients. As a result, total neoadjuvant FLOT (delivering all eight cycles of FLOT preoperatively without postoperative therapy) may be a viable alternative [44, 45], although this approach should not compromise the patient’s ability to undergo surgery due to cumulative chemotherapy toxicities. Further research is needed to establish a new framework for moving forward with personalised perioperative systemic therapy in oesophagogastric cancer, incorporating both pathological and molecular assessments.

## Supplementary Information

Below is the link to the electronic supplementary material.

Supplementary file1 (DOCX 478 KB)

## Data Availability

The datasets analysed in the present study are available from the corresponding author upon reasonable request, subject to institutional approvals and adherence to data confidentiality policies.
