# Correction: Impact of perioperative fluorouracil, leucovorin, oxaliplatin, and docetaxel delivery on postoperative survival in locally advanced oesophagogastric adenocarcinoma

**DOI:** 10.1007/s10120-025-01697-5

**Published:** 2025-12-19

**Authors:** Keiji Sugiyama, Sacheen Kumar, Asif Chaudry, Nikhil Patel, Pranav Patel, David Cunningham, Naureen Starling, Sheela Rao, Charlotte Fribbens, Ian Chau

**Affiliations:** 1 Gastrointestinal Unit, Department of Medicine, The Royal Marsden Hospital, Downs Road, Sutton, London, Surrey UK; 2 Department of Medical Oncology, NHO Nagoya Medical Center, Nagoya, Aichi Japan; 3 Department of Upper Gastrointestinal Surgery, The Royal Marsden Hospital, London, UK

**Correction to: Gastric Cancer (2025) 28:968–981** 10.1007/s10120-025-01643-5

In this article, references 31 to 40 were incorrectly assigned and did not correspond to the intended citations. These have been corrected in this correction.

In the sentence beginning “Previously, at least 15 retrospective studies evaluated …” in this article, the ‘supplementary material 6’ citation ‘10–12, 19–30’ should have read ‘10–12, 22, 23, 25–32, 35, 36’.

The original article has been corrected.

**References (corrected)**

31. Sisic L, Blank S, Nienhüser H, Haag GM, Jäger D, Bruckner T, et al. The postoperative part of perioperative chemotherapy fails to provide a survival benefit in completely resected esophagogastric adenocarcinoma. Surg Oncol. 2020;33:177–88.
32. Papaxoinis G, Kamposioras K, Weaver JMJ, Kordatou Z, Stamatopoulou S, Germetaki T, et al. The role of continuing perioperative chemotherapy post surgery in patients with esophageal or gastroesophageal junction adenocarcinoma: a multicenter cohort study. J Gastrointest Surg. 2019;23:1729–41.
33. Cortes-Mejia NA, Lillemoe HA, Cata JP. Return to intended oncological therapy: state of the art and perspectives. Curr Oncol Rep. 2024;26:1420–30.
34. Koo A, Mavani PT, Sok C, Goyal S, Concors S, Mason MC, et al. Effect of minimally invasive gastrectomy on return to intended oncologic therapy for gastric cancer. Ann Surg Oncol. 2025;32:230–9.
35. Saunders JH, Bowman CR, Reece-Smith AM, Pang V, Dorrington MS, Mumtaz E, et al. The role of adjuvant platinum-based chemotherapy in esophagogastric cancer patients who received neoadjuvant chemotherapy prior to definitive surgery. J Surg Oncol. 2017;115:821–9.
36. Glatz T, Bronsert P, Schäfer M, Kulemann B, Marjanovic G, Sick O, et al. Perioperative platin-based chemotherapy for locally advanced esophagogastric adenocarcinoma: postoperative chemotherapy has a substantial impact on outcome. Eur J Surg Oncol. 2015;41:1300–7.
37. Athauda A, Nankivell M, Langer R, Pritchard S, Langley RE, von Loga K, et al. Pathological regression of primary tumour and metastatic lymph nodes following chemotherapy in resectable OG cancer: pooled analysis of two trials. Br J Cancer. 2023;128:2036–43.
38. Smyth EC, Fassan M, Cunningham D, Allum WH, Okines AFC, Lampis A, et al. Effect of pathologic tumor response and nodal status on survival in the Medical Research Council Adjuvant Gastric Infusional Chemotherapy trial. J Clin Oncol. 2016;34:2721–7.
39. Huffman BM, Aushev VN, Budde GL, Chao J, Dayyani F, Hanna D, et al. Analysis of circulating tumor DNA to predict risk of recurrence in patients with esophageal and gastric cancers. JCO Precis Oncol. 2022;6:e2200420.
40. Hoeppner J, Brunner T, Schmoor C, Bronsert P, Kulemann B, Claus R, et al. Perioperative chemotherapy or preoperative chemoradiotherapy in esophageal cancer. N Engl J Med. 2025;392:323–35.

