# Supplementary material for: Impact of perioperative fluorouracil, leucovorin, oxaliplatin, and docetaxel delivery on postoperative survival in locally advanced oesophagogastric adenocarcinoma
Source: Gastric Cancer. 2025 Jul 14;28(5):968–81. doi: 10.1007/s10120-025-01643-5 (PMC12378138; doi:10.1007/s10120-025-01643-5)
Supplement: Supplementary file 1 — Supplementary file1 (DOCX 478 KB) [file 10120_2025_1643_MOESM1_ESM.docx]

**Supplementary material**

**Impact of Perioperative Fluorouracil, Leucovorin, Oxaliplatin, and Docetaxel Delivery on Postoperative Survival in Locally Advanced Oesophagogastric Adenocarcinoma**

**Journal name:** *Gastric Cancer*

Keiji Sugiyama, Sacheen Kumar, Asif Chaudry, Nikhil Patel, Pranav Patel, David Cunningham, Naureen Starling, Sheela Rao, Charlotte Fribbens, and Ian Chau

**Corresponding Author**:

Professor Ian Chau

Department of Medicine, Royal Marsden Hospital, Downs Road, Sutton, Surrey, United Kingdom SM2 5PT

Phone Number: +44 208 915 6196

Email address: [ian.chau@rmh.nhs.uk](mailto:ian.chau@rmh.nhs.uk)

Supplementary Material 1. Post hoc power analyses

|  | Power |
| --- | --- |
| Peri-operative therapy completion: 3-year differences | |
| Before propensity score matching  OS  RFS | 44%  10% |
| After propensity score matching  OS  RFS | 46%  11% |
| Adjuvant therapy use: 3-year differences | |
| Before propensity score matching  OS  RFS | 50%  51.6% |
| After propensity score matching  OS  RFS | 92%  55.9% |
| Nodal status 3-year differences | |
| Pathological node negative  OS  RFS | 4%  3% |
| Pathological node positive  OS  PFS | 18%  38% |
| Tumour regression grade (2 categories) 3-year differences | |
| TRG 1-2  OS  RFS | 31%  10% |
| TRG 3-5  OS  RFS | 45%  62% |
| Tumour regression grade (3 categories) 3-year differences | |
| TRG 1  OS  RFS | 48%  37% |
| TRG 2-4  OS  RFS | 16%  21% |
| TRG 5  OS  RFS | 46%  59% |

OS: Overall survival; RFS: relapse free survival; TRG: tumour regression grade

**Supplementary Material 2.** Study schema


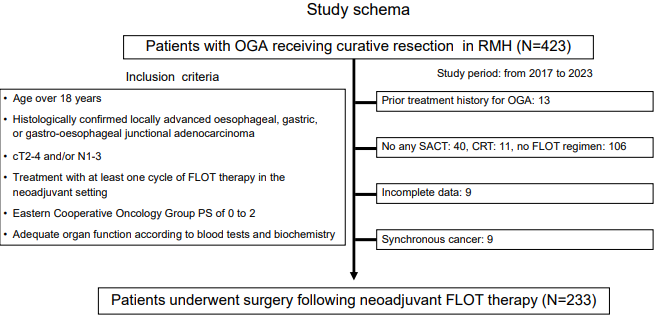


Flowchart depicting patient selection and disposition. CRT, chemoradiotherapy; CTx, chemotherapy; FLOT, 5-fluorouracil, leucovorin, oxaliplatin, docetaxel; OGA, oesophagogastric adenocarcinoma; PS, performance status; SACT, systemic anti-cancer treatment

**Supplementary Material 3. Summary of treatment exposure of perioperative FLOT. (a): Prevalence of perioperative FLOT delivery, specifically the completion of neoadjuvant chemotherapy, receipt of adjuvant chemotherapy, completion of adjuvant chemotherapy, and perioperative chemotherapy completion in the entire cohort and patients treated with oesophagectomy and gastrectomy. (b): Histogram representing the number of chemotherapy cycles administered during the perioperative period.**


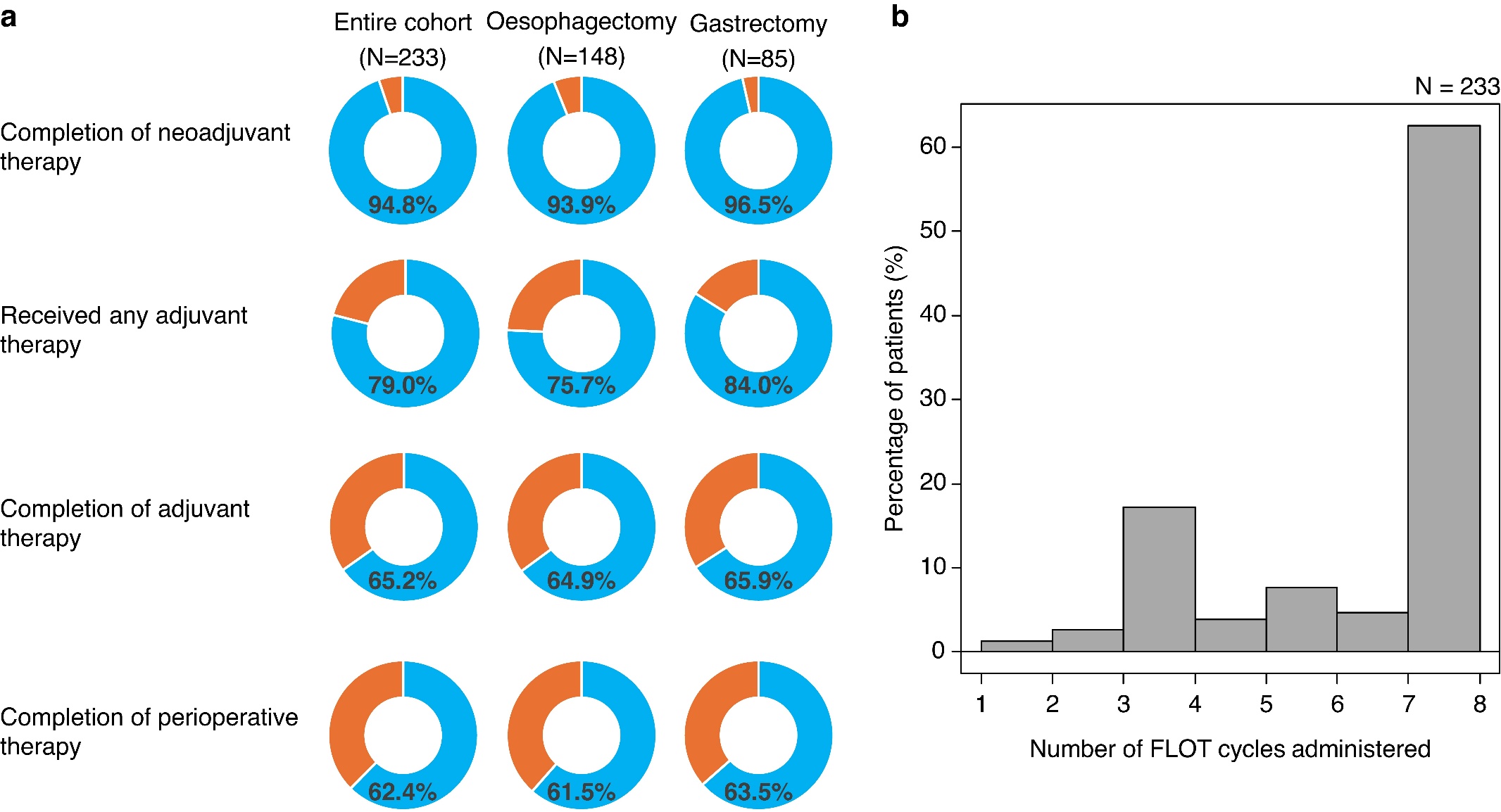


**FLOT, 5-fluorouracil, leucovorin, oxaliplatin, docetaxel**

**Supplementary Material 4.** Baseline characteristics of the patients a) before and after PSM. PSM was performed for patients who completed perioperative FLOT vs those who did not and patients who received adjuvant chemotherapy vs those who did not, b) pathological nodal negative and positive group, c) TRG1, 2–4 and group.

a)

| **Characteristics** |  | **Before PSM** | | | | **After PSM** | | | | **Before PSM** | | | | **After PSM** | | | |
| --- | --- | --- | --- | --- | --- | --- | --- | --- | --- | --- | --- | --- | --- | --- | --- | --- | --- |
|  |  | **Tx comp (N=145)** | **Tx incomp (N=88)** | ***p*-value** | **SMD** | **Tx comp (N=78)** | **Tx incomp (N=78)** | ***p*-value** | **SMD** | **ACT+ (N=49)** | **ACT- (N=184)** | ***p*-value** | **SMD** | **ACT+ (N=45)** | **ACT- (N=45)** | ***p*-value** | **SMD** |
| Age (year), mean (SD) |  | 62.45 (10.58) | 64.36 (10.49) | 0.18 | 0.18 | 64.66 (9.13) | 63.86 (10.47) | 0.61 | 0.08 | 65.65 (10.49) | 62.51 (10.51)/ 41 (22.3) | 0.06 | 0.29 | 63.43 (9.36) | 65.70 (10.84) | 0.28 | 0.22 |
| Sex | Female | 34 (23.4) | 16 (18.2) | 0.41 | 0.13 | 15 (19.2) | 16 (20.5) | 1 | 0.03 | 9 (18.4) | 43  (22.3) | 0.69 | 0.09 | 8 (17.8) | 9 (20.0) | 1 | 0.05 |
|  | Male | 111 (76.6) | 72 (81.8) |  |  | 63 (80.8) | 62 (79.5) |  |  | 40 (81.6) | 143 (77.7) |  |  | 37 (82.2) | 36 (80.0) |  |  |
| ECOG-PS | 0 | 91 (62.8) | 43 (48.9) | 0.04 | 0.28 | 43 (55.1) | 39 (50.0) | 0.63 | 0.1 | 27 (55.1) | 107 (58.2) | 0.74 | 0.06 | 32 (71.1) | 24 (53.3) | 0.12 | 0.37 |
|  | 1–2 | 54 (37.2) | 45 (51.1) |  |  | 35 (44.9) | 35 (44.9) |  |  | 22 (44.9) | 77 (41.8) |  |  | 13 (28.9) | 21 (46.7) |  |  |
| BMI (kg/m^2^), mean (SD) |  | 27.05 (4.90) | 26.70 (4.97) | 0.59 | 0.07 | 27.01 (5.34) | 26.72 (5.01) | 0.72 | 0.05 | 25.86 [17.67, 40.89] | 26.54 [16.10, 46.10] | 0.56 | 0.08 | 26.85 (5.38) | 26.61 (5.23) | 0.83 | 0.04 |
| Weight loss | <5% | 72  (49.7) | 39 (44.3) | 0.49 | 0.1 | 35 (44.9) | 33 (42.3) | 0.87 | 0.05 | 22 (44.9) | 89 (48.4) | 0.74 | 0.07 | 17 (37.8) | 18 (40.0) | 1 | 0.04 |
|  | ≥5% | 3 (50.3) | 49 (55.7) |  |  | 43 (55.1) | 45 (57.7) |  |  | 27 (55.1) | 95 (51.6) |  |  | 28 (62.2) | 27 (60.0) |  |  |
| Histology | Differentiated or  Mixed | 84 (57.9) | 56 (63.6) | 0.41 | 0.11 | 47 (60.3) | 49 (62.8) | 0.86 | 0.05 | 30 (61.2) | 110 (59.8) | 1 | 0.02 | 23 (51.1) | 26 (57.8) | 0.67 | 0.13 |
|  | Poorly differentiated | 61 (42.1) | 32 (36.4) |  |  | 31 (39.7) | 29 (37.2) |  |  | 19 (38.8) | 4 (40.2) |  |  | 22  (48.9) | 19 (42.2) |  |  |
| MMR status | pMMR | 117 (80.7) | 71 (80.7) | 0.43 | 0.2 | 68 (87.2) | 63 (80.8) | 0.433 | 0.23 | 37 (75.5) | 151 (82.1) | 0.41 | 0.21 | 35 (77.8) | 35 (77.8) | 1 | <0.001 |
|  | dMMR | 6  (4.1) | 1  (1.1) |  |  | 2  (2.6) | 1  (1.3) |  |  | 1  (2.0) | 6  (3.3) |  |  | 1  (2.2) | 1  (2.2) |  |  |
|  | NA | 22 (15.2) | 16 (18.2) |  |  | 8 (10.3) | 14 (17.9) |  |  | 11 (22.4) | 27 (14.7) |  |  | 9 (20.0) | 9 (20.0) |  |  |
| Tumour location | Oesophagus | 72 (49.7) | 46 (52.3) | 0.93 | 0.05 | 40 (51.3) | 40 (51.3) | 0.79 | 0.11 | 30 (61.2) | 88 (47.8) | 0.12 | 0.34 | 27 (60.0) | 28 (62.2) | 0.91 | 0.11 |
|  | OGJ | 29 (20.0) | 17 (19.3) |  |  | 19 (24.4) | 16 (20.5) |  |  | 10 (20.4) | 36 (19.6) |  |  | 8 (17.8) | 9 (20.0) |  |  |
|  | Gastric | 44 (30.3) | 25 (28.4) |  |  | 19 (24.4) | 22 (28.2) |  |  | 9 (18.4) | 60 (32.6) |  |  | 10 (20.2) | 8 (17.8) |  |  |
| cT stage | T1–2 | 27 (18.9) | 22 (25.3) | 0.25 | 0.15 | 21 (26.9) | 19 (24.4) | 0.85 | 0.05 | 13 (26.5) | 36 (19.9) | 0.32 | 0.15 | 13 (28.9) | 12 (26.7) | 1 | 0.05 |
|  | T3–4 | 116 (81.1) | 65 (74.7) |  |  | 57 (73.1) | 59 (75.6) |  |  | 36 (73.5) | 145 (80.1) |  |  | 32 (71.1) | 33 (73.3) |  |  |
| cN stage | N0 | 52 (35.9) | 40 (45.5) | 0.16 | 0.19 | 33 (42.3) | 35 (44.9) | 0.87 | 0.05 | 21 (42.9) | 71 (38.6) | 0.62 | 0.08 | 26 (57.8) | 21 (46.7) | 0.39 | 0.22 |
|  | N+ | 93 (64.1) | 48 (54.5) |  |  | 45 (57.7) | 43 (55.1) |  |  | 28 (57.1) | 113 (61.4) |  |  | 19 (42.2) | 24 (53.3) |  |  |
| NLR, mean (SD) |  | 2.84 (1.15) | 2.88 (1.65) | 0.79 | 0.03 | 2.94 (1.22) | 2.85 (1.69) | 0.7 | 0.06 | 3.03 (1.67) | 2.81 (1.27) | 0.31 | 0.15 | 2.95 (1.19) | 3.01 (1.68) | 0.86 | 0.03 |
| Alb (g/dL) (SD) |  | 41.94 (4.11) | 42.10 (4.41) | 0.79 | 0.03 | 42.14 (4.35) | 42.04 (4.53) | 0.88 | 0.02 | 41.94 (4.11) | 42.10 (4.41) | 0.79 | 0.03 | 41.04 (4.56) | 42.07 (4.37) | 0.28 | 0.22 |
| CKD | ≥60 | 70 (49.0) | 38 (45.2) | 0.68 | 0.07 | 40 (51.3) | 38 (48.7) | 0.87 | 0.05 | 23 (51.1) | 85 (46.7) | 0.62 | 0.08 | 24 (53.3) | 23 (51.1) | 1 | 0.04 |
|  | <60 | 73 (51.0) | 46 (54.8) |  |  | 38 (48.7) | 40 (51.3) |  |  | 22 (48.9) | 97 (53.3) |  |  | 21 (46.7) | 22 (48.9) |  |  |

b)

| **Characteristics** |  | **ypN negative** | | | | **ypN positive** | | | |
| --- | --- | --- | --- | --- | --- | --- | --- | --- | --- |
|  |  | **ACT+**  **(N=105)** | **ACT-**  **(N=22)** | ***p*-value** | **SMD** | **ACT+**  **(N=77)** | **ACT-**  **(N=29)** | ***p*-value** | **SMD** |
| Age (year), mean (SD) |  | 62.74 (10.38) | 68.83 (9.93) | 0.013 | 0.6 | 62.11 (10.86) | 63.29 (10.08) | 0.611 | 0.113 |
| Sex | Female | 21 (20.0) | 4 (18.2) | 1 | 0.046 | 20 (26.0) | 5 (17.2) | 0.445 | 0.213 |
|  | Male | 84 (80.0) | 18 (81.8) |  |  | 57 (74.0) | 24 (82.8) |  |  |
| ECOG-PS | 0 | 64 (61.0) | 14 (63.6) | 1 | 0.055 | 41 (53.9) | 14 (48.3) | 0.665 | 0.114 |
|  | 1–2 | 41 (39.0) | 8 (36.4) |  |  | 35 (46.1) | 15 (51.7) |  |  |
| BMI (kg/m^2^), mean (SD) |  | 27.32 (4.73) | 26.44 (4.87) | 0.433 | 0.183 | 26.60 (5.10) | 26.69 (5.27) | 0.937 | 0.017 |
| Weight loss | <5% | 56 (53.3) | 11 (50.0) | 0.818 | 0.067 | 32 (41.6) | 12 (41.4) | 1 | 0.004 |
|  | ≥5% | 49 (46.7) | 11 (50.0) |  |  | 45 (58.4) | 17 (58.6) |  |  |
| Histology | Differentiated or mixed | 65 (63.1) | 12 (57.1) | 0.628 | 0.122 | 42 (54.5) | 14 (48.3) | 0.664 | 0.126 |
|  | Poorly differentiated | 38 (36.9) | 9 (42.9) |  |  | 35 (45.5) | 15 (51.7) |  |  |
| MMR status | pMMR | 47 (44.8) | 10 (45.5) | 1 | 0.014 | 71 (92.2) | 24 (82.8) | 0.175 | 0.448 |
|  | dMMR | 58 (55.2) | 12 (54.5) |  |  | 2 (2.6) | 0 (0.0) |  |  |
|  | NA | 23 (22.5) | 6 (27.3) | 0.592 | 0.109 | 4 (5.2) | 5 (17.2) |  |  |
| Tumour location | Oesophagus | 79 (77.5) | 16 (72.7) |  |  | 37 (48.1) | 16 (55.2) | 0.406 | 0.302 |
|  | OGJ | 79 (75.2) | 14 (63.6) | 0.423 | 0.258 | 14 (18.2) | 7 (24.1) |  |  |
|  | Gastric | 4 (3.8) | 1 (4.5) |  |  | 26 (33.8) | 6 (20.7) |  |  |
| cT stage | T1–2 | 22 (21.0) | 7 (31.8) |  |  | 13 (16.9) | 7 (24.1) | 0.412 | 0.18 |
|  | T3–4 | 68 (64.8) | 16 (72.7) | 0.622 | 0.172 | 64 (83.1) | 22 (75.9) |  |  |
| cN stage | N0 | 37 (35.2) | 6 (27.3) |  |  | 24 (31.2) | 11 (37.9) | 0.644 | 0.143 |
|  | N+ | 51 (48.6) | 14 (63.6) | 0.429 | 0.341 | 53 (68.8) | 18 (62.1) |  |  |
| NLR, mean (SD) |  | 21 (20.0) | 4 (18.2) |  |  | 2.74 (1.10) | 3.07 (1.75) | 0.253 | 0.228 |
| Alb (g/dL) (SD) |  | 33 (31.4) | 4 (18.2) |  |  | 42.01 (4.16) | 41.46 (4.38) | 0.565 | 0.129 |
| CKD | ≥60 | 42.01 (4.16) | 42.57 (4.70) | 0.582 | 0.127 | 41 (53.2) | 14 (53.8) | 1 | 0.162 |
|  | <60 | 2.86 (1.38) | 2.99 (1.54) | 0.704 | 0.088 | 36 (46.8) | 12 (46.2) |  |  |

c)

| **Characteristics** |  | **TRG1** | | | | **TRG2–4** | | | | **TRG5** | | | |
| --- | --- | --- | --- | --- | --- | --- | --- | --- | --- | --- | --- | --- | --- |
|  |  | **ACT+ (N=32)** | **ACT- (N=9)** | ***p*-value** | **SMD** | **ACT+ (N=103)** | **ACT- (N=28)** | ***p*-value** | **SMD** | **ACT+ (N=26)** | **ACT- (N=12)** | ***p*-**  **value** | **SMD** |
| Age (year), mean (SD) |  | 63.64 (9.70) | 69.23 (9.87) | 0.136 | 0.571 | 62.02 (10.10) | 65.53 (10.48) | 0.108 | 0.341 | 60.34 (13.29) | 63.23 (11.05) | 0.518 | 0.236 |
| Sex | Female | 4 (12.5) | 1 (11.1) | 1 | 0.043 | 22 (21.4) | 8 (28.6) | 0.451 | 0.167 | 7 (26.9) | 0 (0.0) | 0.074 | 0.858 |
|  | Male | 28 (87.5) | 8 (88.9) |  |  | 81 (78.6) | 20 (71.4) |  |  | 19 (73.1) | 12 (100.0) |  |  |
| ECOG-PS | 0 | 18 (56.2) | 5 (55.6) | 1 | 0.014 | 60 (58.3) | 16 (57.1) | 0.279 | 0.273 | 14 (53.8) | 6 (50.0) | 1 | 0.077 |
|  | 1–2 | 14 (43.8) | 4 (44.4) |  |  | 43 (41.7) | 12 (42.9) |  |  | 12 (46.2) | 6 ( 50.0) |  |  |
| BMI (kg/m^2^), mean (SD) |  | 27.38 (4.30) | 23.87 (3.45) | 0.031 | 0.899 | 27.34 (5.12) | 27.21 (5.78) | 0.912 | 0.023 | 25.18 (5.00) | 27.17 (4.18) | 0.238 | 0.433 |
| Weight loss | <5% | 16 (50.0) | 6 (66.7) | 0.466 | 0.343 | 49 (47.6) | 10 (35.7) | 0.291 | 0.242 | 15 (57.7) | 6 (50.0) | 0.734 | 0.155 |
|  | ≥5% | 16 (50.0) | 3 (33.3) |  |  | 54 (52.4) | 18 (64.3) |  |  | 11 (42.3) | 6 (50.0) |  |  |
| Histology | Differentiated  or mixed | 18 (56.2) | 8 (88.9) | 0.119 | 0.786 | 67 (65.0) | 17 (60.7) | 0.664 | 0.09 | 10 (38.5) | 5 (41.7) | 1 | 0.065 |
|  | Poorly differentiated | 14 (43.8) | 1 (11.1) |  |  | 36 (35.0) | 11 (39.3) |  |  | 16 (61.5) | 7 (58.3) |  |  |
| MMR status | pMMR | 20 (62.5) | 6 (66.7) | 1 | 0.258 | 92 (89.3) | 20 (71.4) | 0.023 | 0.515 | 20 (76.9) | 11 (91.7) | 0.395 | 0.414 |
|  | dMMR | 1  (3.1) | 0 (0.0) |  |  | 4 (3.9) | 1 (3.6) |  |  | 0 (0) | 0 (0) |  |  |
|  | NA | 11 (34.4) | 3 (33.3) |  |  | 7 (6.8) | 7 (25.0) |  |  | 6 (23.1) | 1 (8.3) |  |  |
| Tumour location | Oesophagus | 15 (46.9) | 6 (66.7) | 0.618 | 0.446 | 56 (54.4) | 18 (64.3) | 0.572 | 0.245 | 9 (34.6) | 6 (50.0) | 0.226 | 0.674 |
|  | OGJ | 9 (28.1) | 2 (22.2) |  |  | 14 (13.6) | 4 (14.3) |  |  | 5 (19.2) | 4 (33.3) |  |  |
|  | Gastric | 8 (25.0) | 1 (11.1) |  |  | 33 (32.0) | 6 (21.4) |  |  | 12 (46.2) | 2 (16.7) |  |  |
| cT stage | T1–2 | 4 (12.9) | 1 (11.1) | 1 | 0.055 | 18 (17.5) | 8 (28.6) | 0.193 | 0.266 | 7 (28.0) | 4 (33.3) | 1 | 0.116 |
|  | T3–4 | 27 (87.1) | 8 (88.9) |  |  | 85 (82.5) | 20 (71.4) |  |  | 18 (72.0) | 8 (66.7) |  |  |
| cN stage | N0 | 9 (28.1) | 4 (44.4) | 0.429 | 0.344 | 40 (38.8) | 10 (35.7) | 0.829 | 0.065 | 12 (46.2) | 7 (58.3) | 0.728 | 0.246 |
|  | N+ | 23 (71.9) | 5 (55.6) |  |  | 63 (61.2) | 18 (64.3) |  |  | 14 (53.8) | 5 (41.7) |  |  |
| NLR, mean (SD) |  | 2.67 (1.17) | 3.58 (2.00) | 0.098 | 0.558 | 2.72 (1.07) | 2.49 (1.06) | 0.316 | 0.221 | 2.85 (1.15) | 3.85 (2.15) | 0.069 | 0.58 |
| Alb (g/dL) (SD) |  | 41.94 (4.04) | 42.00 (5.86) | 0.972 | 0.012 | 42.48 (3.98) | 42.72 (4.12) | 0.789 | 0.059 | 41.04 (3.36) | 40.75 (3.82) | 0.815 | 0.08 |
| CKD | ≥60 | 24 (75.0) | 5 (62.5) | 0.66 | 0.272 | 53 (52.0) | 12 (48.0) | 0.824 | 0.079 | 11 (42.3) | 8 (66.7) | 0.295 | 0.504 |
|  | <60 | 8 (25.0) | 3 (37.5) |  |  | 49 (48.0) | 13 (52.0) |  |  | 15 (57.7) | 4 (33.3) |  |  |

**Supplementary Material 5**. Restricted Mean Survival Time differences at 36 months

| Comparison Group | OS RMST Difference, months (95%CI) | RFS RMST Difference, months (95%CI) |
| --- | --- | --- |
| Perioperative therapy Completion vs Non-completion (All patients, PSM data) | 3.9 (0.2–7.6), p=0.03 | 3.2 (-1.2–7.6), p=0.15) |
| ACT vs No ACT (All patients, PSM data) | 8.8 (4.2–13.4), p<0.01 | 9.7 (-4.3–15.1), p=<0.01 |
| ACT vs No ACT (ypN-positive, unadjusted data) | 8.2 (1.0–15.3), p=0.02 | 7.2 (-0.2–14.6), p=<0.001 |
| ACT vs No ACT (ypN-negative, unadjusted data) | 0.1 (-4.2–4.4), p=0.95 | 3.6 (-2.7–10.1), p=0.26 |

ACT, Adjuvant Chemotherapy; OS, overall survival; PSM, propensity score matching; RFS, recurrence-free survival; RMST, Restricted Mean Survival Time.

**Online Resource 6**. Summary of previous studies that compared neoadjuvant and perioperative therapies for locally advanced oesophagogastric adenocarcinoma

|  | Country | Multicentre | Study period | Tumour location (oesophagus/gastro-oesophageal junction/gastric) | Regimen | Patients, N | % of patients who received ACT | OS benefit with ACT (OS in those who received ACT vs. NAC alone) | Remarkable findings in subgroup analysis | PSM applied |
| --- | --- | --- | --- | --- | --- | --- | --- | --- | --- | --- |
| Present study | UK | No | 2017–2023 | 50.6%/19.7%/29.6% | FLOT (100%) | 233 | 79.0% | Yes (3y-OS rate: 66% vs. 53%, *p*=0.02) | Survival benefit was observed in the ypN+ subgroup and across the HPR but not in the ypN- subgroup | Yes |
| MMirza et al., 2013[41] | UK | No | 1996–2010 | -/64%/ 36% | ECF | 66 | 47.0% | Yes (*p*=0.02, HR: 0.26, *p*=0.008) |  |  |
| Luc et al., 2015[42] | France | No | 2000–2012 | 18%/43%/ 39% | ECF | 110 | 67.0% | Yes (HR: 5.13, 95% CI 1.55–16.97, *p*=0.007) |  |  |
| GGlatz et al., 2015[30] | Germany | No | 2006–2013 | 72%/-/28% | ECF/EOX (43%), FLOT (57%) | 134 | 64.0% | Yes (5-y OS: 75.8% vs. 40.3%, *p*<0.01) | Improved OS in patients with ypN+ (5y-OS: 64.5% vs. 9.7%, *p*=0.002) and poor HPR (55.5% vs. 19.3%, *p*=0.015) |  |
| Lichthardt et al., 2016[43] | Germany | No | 2006–2013 | 42%/57%/- | ECX/ECF/FLOT | 72 | 72.0% | No (3y-OS: 31.4% vs. 76.3%, *p*<0.01) |  |  |
| Saunders et al., 2017[29] | UK | No | 2006–2013 | 35%/47%/ 17% | ECF/ECX | 333 | 57.0% | Not described for the entire cohort | Improved OS in patients with HPR (HR 0.51, 95% CI 0.28–0.93, *p*=0.028) but not in the non-responding group. |  |
| Sisic et al., 2020[21] | Germany | No | 2006–2015 | 62%/-/ 38% | ECX (46%), PF/FLO/OX (17%), FLOT (36%) | 299 | 57.0% | No (mOS: 78.2 months vs. not reached, *p*=0.33) | Improved RFS in non-intestinal tumours (*p*=0.023) and patients receiving the FLOT regimen (*p*=0.038) |  |
| Karagkonunis et al., 2017[44] | USA | YYes | 2000–2012 | 23%/73%/- | ECX (79%) | 163 | 69.0% | Yes (HR: 0.33, 95% CI: 0.14–0.82, *p*=0.01) |  |  |
| Papaxoinis et al., 2019[22] | UK | YYes | 2009–2017 | 33%/67% | ECX (99%) | 312 | 72.0% | No (mOS: 46.1 vs. 36.7 months, *p*=0.19) | Improved OS (HR 0.53, 95% CI 0.31–0.90, *p*=0.018) in patients with R1 resection | Yes |
| Van Putten et al., 2019[45] | The Netherlands | YYes | 2006–2014 | -/-/100% | n.a. | 1686 | 57.0% | Yes (HR: 0.84, 95% CI: 0.71–0.99) |  | YYes |
| Coimbra et al., 2019[46] | Brazil | NNo | 2006–2016 | -/-/100% | DCF/DCX (30%), PF (59%) | 225 | 65.0% | Yes (5-y OS: 70.3% vs. 59.9%, HR 0.55, 95% CI: 0.33–0.91) |  |  |
| Drake et al., 2021[10] | USA | Yes | 2006–2014 | -/-/100% | n.a. | 3449 | 32.0% | No (mOS: 56.8 vs. 52.5 months, *p*=0.13) | Improved OS in patients with ypN+ (79.6 vs. 41.3 months, *p*=0.025) | YYes |
| Deng et al., 2021[12] | USA | Yes | 2006–2017 | -/-/100% | n.a. | 2382 | 36% | No (HR: 0.88, 95% CI: 0.75–1.02) | Improved 5-year survival in patients with response to NAC (73.8% vs. 65.0%; HR 0.64, 95% CI 0.46–0.91, *p*=0.02) but not those with ypT0N0 or non-responding disease (pTNM ≥cTNM) | YYes |
| Rahman et al., 2022[11] | UK | Yes | 2012–2018 | 47.9%/29.2%/22.9% | ECF/ECX (96.7%), FLOT (3.3%) | 4139 | 38.5% | Yes (mOS 62.7 vs. 50.4 months, HR: 0.84, 95% CI: 0.77–0.94, *p*=0.001) | Improved OS in the ypN+ subgroup (HR: 0.80, 95% CI: 0.70–0.92, *p*<0.001) but not in the ypN- subgroup | YYes |
| Ballhause et al., 2022[28] | Germany | Yes | 2006–2010 | 10%/45.5%/44.5% | ECX/ECF (55.5%), DCF/DCX (44.5%) | 110 | 58.0% | Yes (mOS 35.7 vs. 19.2 months, *p*=0.002) | No significant benefit was observed in patients with ypN+ |  |
| Lin et al., 2022[27] | International | Yes | 2008–2017 | -/-/100% | CAPOX/SOX, FOLFOX, ECF/ECX | 353 | 74.0% | Yes (3y-OS: 60.1% vs. 49.3%, *p*=0.02) | Improved OS in patients with an LNR ≥9% (OS: HR: 0.45; 95% CI: 0.29–0.69, *p*=0.007) but not in those with an LNR <9% | YYes |

ACT, Adjuvant Chemotherapy; DCF, Docetaxel, Cisplatin, and Fluorouracil; DCX, Docetaxel, Cisplatin, and Capecitabine; ECF, Epirubicin, Cisplatin, and Fluorouracil; ECX, Epirubicin, Cisplatin, and Capecitabine; EOX, Epirubicin, Oxaliplatin, and Capecitabine; FLOT, 5-Fluorouracil, Leucovorin, Oxaliplatin, and Docetaxel; HPR, Histopathological response; HR, Hazard ratio; OS, Overall survival; PSM, Propensity score matching; RFS, Recurrence-Free Survival
